# Pre-pregnancy body surface area and risk for gestational diabetes mellitus

**DOI:** 10.1007/s00592-022-02029-0

**Published:** 2023-01-13

**Authors:** Lotta S. Holopainen, Hanna H. Tähtinen, Mika Gissler, Päivi E. Korhonen, Mikael O. Ekblad

**Affiliations:** 1 Department of General Practice, Institute of Clinical Medicine, University of Turku and Turku University Hospital, Turun Yliopisto, Yleislääketiede, 20014 Turku, Finland; 2 Department of Knowledge Brokers, THL Finnish Institute for Health and Welfare, Helsinki, Finland; 3 Research Centre for Child Psychiatry, University of Turku, Turku, Finland; 4 Department of Molecular Medicine and Surgery, Karolinska Institute, Stockholm, Sweden; 5 Region Stockholm, Academic Primary Health Care Centre, Stockholm, Sweden

**Keywords:** Gestational diabetes, Body surface area, Body size, Smoking, Pregnancy

## Abstract

**Aims:**

To evaluate the effect of the pre-pregnancy body surface area (BSA) on the risk of gestational diabetes mellitus (GDM).

**Methods:**

The study population consisted of all primiparous women with singleton pregnancies (*n* = 328,892) without previously diagnosed diabetes or chronic hypertension in Finland between 2006 and 2019. The information on GDM, oral glucose tolerance test (OGTT) results, and maternal backgrounds was derived from the Finnish Medical Birth Register. The pre-pregnancy BSA was calculated by using the Mosteller formula. Logistic regression models were used to estimate the association between BSA and GDM/ OGTT separately by the body mass index groups.

**Results:**

A lower BSA predicted an increased risk for GDM and pathological OGTT among the underweight (*b* = − 2.69, SE = 0.25, *p* < 0.001; *b* = − 2.66, SE = 0.23, *p* < 0.001, respectively) pregnant women, and normal weight (*b* = − 0.30, SE = 0.10, *p* = 0.002; *b* = − 0.67, SE = 0.09, *p* < 0.001, respectively) pregnant women; and pathological OGTT among the overweight (*b* = − 0.31, SE = 0.10, *p* = 0.001) pregnant women. Within the obese class II or greater, a higher BSA predicted a higher risk for GDM (*b* = 0.74, SE = 0.12, *p* < 0.001) and pathological OGTT (*b* = 0.79, SE = 0.13, *p* < 0.001). Maternal smoking predicted a significantly higher risk of GDM and pathological OGTTs in almost all body mass index groups.

**Conclusion:**

This study showed that in comparison with women with a higher BSA, underweight, and normal weight pregnant women with a smaller BSA may be more susceptible to GDM and have a pathological OGTT.

**Supplementary Information:**

The online version contains supplementary material available at 10.1007/s00592-022-02029-0.

## Introduction

Gestational diabetes mellitus (GDM) is a pregnancy complication, in which glucose metabolism is impaired due to the pregnancy-induced pancreatic B-cell dysfunction and insulin resistance. Altered glucose metabolism occurs for the first time during pregnancy, and in most cases, it disappears after childbirth. The risk of fetal macrosomia increases with GDM, predisposing the pregnant woman to complications during labor. People with GDM have a greater risk of gestational hypertension and pre-eclampsia than healthy parturients [1]. GDM also substantially increases the risk for future maternal health problems, e.g., cardiovascular events and type 2 diabetes [2]. The main risks for GDM are maternal obesity, previously diagnosed non-alcoholic liver disease, older age, and family history of any type of diabetes [3, 4]. The percentage of pre-pregnancy overweight (BMI ≥ 25) women was 41.9% and obese (BMI ≥ 30) women were 17.0% in Finland in 2020 [5]. GDM is an increasingly common condition during pregnancy. In the year 2019, GDM was diagnosed in 19.1% of Finnish mothers during their pregnancy. The prevalence and the diagnostic criteria for GDM varies globally [6]. In Finland, gestational diabetes is diagnosed if one of the following events occurs: fasting plasma glucose level > 5.3 mmol/l, one hour after a 75 g glucose dose > 10.0 mmol/l, or two hours after a glucose dose > 8.6 mmol/l [1].

The national Finnish Current Care Guidelines for GDM recommends screening almost all pregnant women, except for those with a low risk for GDM, during their first pregnancy for GDM [1]. Gestational diabetes is diagnosed with an oral glucose tolerance test (OGTT). The OGTT consists of a uniform glucose dose of 75 g to all pregnant women regardless of their body size [7]. A reverse association between the post load 2-h plasma glucose and body height has been demonstrated in several studies in the general population, as well as among pregnant women [8–10]. It has also been found that shorter pregnant women are more susceptible to a GDM diagnosis [10].

Body surface area (BSA) is a total surface area of the human body, and it is commonly used to evaluate drug doses and medical indicators or estimates [11]. It has been found that in the non-pregnant population, the body size has a significant effect on the OGTT results. Impaired glucose tolerance was more frequently observed in smaller individuals compared to relatively larger individuals in the general population [8]. The question remains if maternal BSA affects the risk for having a GDM diagnosis.

The aim of this study was to investigate the effect of pre-pregnancy BSA on the risk of GDM among Finnish primiparous women during the years of 2006–2019 by using the population-based register data. We hypothesized that women with a smaller BSA are more susceptible to be diagnosed with GDM compared to women with a higher BSA.

## Methods

### Data sources

The study data sets were drawn from the Finnish Medical Register and the Finnish Hospital Discharge Register. The Finnish Institute for Health and Welfare (THL) performed the ethical review and granted permission to use its confidential register data. To combine all the register data, pregnant women’s personal identity codes were used. Statistical authorities performed the data linkages, and only the unidentifiable data were provided for the researchers outside the Finnish Institute for Health and Welfare.

The Medical Birth Register is considered to be a complete record of all births and newborns in Finland. The register data contain all live births and stillbirths of fetuses with a gestational age of 22 weeks or more or with a birth weight of at least 500 g or more. The register data are collected from all delivery hospitals, and in the case of home births, from the assisting health care personnel. The register contains information on the mother’s and the child’s identity codes; maternal personal data, health care, previous pregnancies and deliveries, and interventions during the pregnancy and delivery; and the newborn’s outcome until it reaches 7 days of age. According to data quality studies, most of the content of the register data corresponds well or satisfactorily with the hospital record data [12].

Information on all episodes of inpatient care, including all hospitalizations requiring an overnight stay in public and private hospitals since 1969 and outpatient visits in public hospitals since 1998, are included in the Hospital Discharge Register. The register includes information on the patient’s background, procedures, hospitalization periods, and the main diagnosis, as well as up to two other diagnoses by the International Classification of Diseases (ICD) code (Eight Revision [ICD-8] in 1969–1986, Ninth Revision [ICD-9] in 1987–1995, and Tenth Revision [ICD-10] since 1996). A systematic review revealed that the completeness and accuracy of the register range from satisfactory to very good [13].

The study data were drawn from the Finnish Medical Register and the Finnish Hospital Discharge Register. The Finnish Institute for Health and Welfare (THL) performed the ethical review and granted the permission to use its confidential register data. As this study a study based on data from a register, informed consent statements for patient enrollment is not applicable. To combine all register data, pregnant women’s personal identity code was used. Statistical authorities performed the data linkages and only unidentifiable data were provided for the researchers outside the Finnish Institute for Health and Welfare.

### Study sample

The study population consisted of all pregnant women with singleton pregnancies (*n* = 835,551) in Finland during the years of 2006–2019. Multiparous women were excluded (*n* = 487,399). The ICD-10 classification was used during the entire study period, and pregnant women with a diagnosis of pre-pregnancy diabetes (ICD-10 codes: O24.0, O24.1, O24.2 and O24.3) were excluded (*n* = 2691). Women with a pre-pregnancy diagnosis of chronic hypertension (*n* = 529) and women without weight and height data (*n* = 16,040) were also excluded. The final study population consisted of 328,892 pregnant women (94.5% of all primiparous women with singleton pregnancies during the study period).

The information on maternal background factors was derived from the Finnish Medical Birth Register. The pre-pregnancy BSA was calculated by using the Mosteller formula; BSA (m^2^) = square root of [(weight (kg) x height (cm))/3600] [14], which utilizes the maternal pre-pregnancy weight and height. Smoking was categorized in three classes: no, quitted in the first trimester, and continued throughout the pregnancy. The BMI was categorized into five groups: < 20 (underweight), 20.0–24.9 (normal weight), 25.0–29.9 (overweight), 30.0–34.9 (obese, class I), and 35 kg/m^2^ or more (obese class II or greater).

### GDM diagnoses

The GDM screening consists of a globally standardized 2-h 75 g OGTT. The national Finnish Current Care Guidelines for GDM recommends screening between 24 and 28 gestational weeks, except for low-risk women with no abnormal glucose tolerance, under 25 years of age, and a pre-pregnancy BMI between 18.5 and 25 kg/m^2^, and women with no family history of diabetes. In women with a higher risk for diabetes, the test is performed earlier between 12 and 16 weeks; and if the test result is normal, the test is repeated between 24 and 28 weeks. If the plasma 12 h fasting glucose level is > 5.3 mmol/l, or after one hour > 10.0 mmol/l, or two hours is > 8.6 mmol/l, the mother is diagnosed with GDM [1].

In this study, GDM diagnoses and information regarding the OGTT were obtained from the Finnish Medical Birth Register. GDM was defined by the ICD-10 codes O24.4 and O24.9. The information regarding OGTT results (normal/abnormal) was found for 177,119 women; for 151,773 women, an OGTT was not performed due to a low risk for GDM or the lack of information.

### Statistics

Logistic regression models were used to estimate the association between BSA and the outcomes. GDM and OGTT were added separately as the independent variable, and BSA as the dependent variable into the model. Maternal age was added as a continuous covariate, and maternal smoking and marital status as a binomial covariate into the model. The analyses were performed separately for the BMI group because the previous studies [4, 8, 10] have shown that shorter or smaller-sized persons may have an increased risk for GDM.

The number of GDM diagnoses was 32,564 (9.9%), and there were more pathological OGTTs, 38,261 (11.6%). We assumed that there was an absence of information on GDM diagnoses in the register data. Thus, we performed sensitivity analyses with the combined information on GDM and pathological OGTTs *(n* = 43,779, 13.3%).

The data analysis was performed with commercially available software (SAS, version 9.4; SAS Institute Inc, Cary, North Carolina). Differences in the results were evaluated by using 95% confidence intervals and p values. Non-overlapping confidence intervals and *P* values < 0.05 were considered to be significant.

## Results

The study included 328,892 Finnish primiparous women with a mean age of 28 years. The mean pre-pregnancy BSA of the study population was 1.73 m^2^ (SD = 0.19). Table 1 presents the characteristics of the participants according to GDM diagnoses and pathological OGTT results. The overall prevalence of GDM in this study cohort was 9.9% (*n* = 32,564). The OGTT was performed for 53.9% (*n* = 177,119) participants, and the pathological OGTT result was observed in 21.6% of those tested. The majority (80.6%) of pregnant women did not smoke during their pregnancy, 7.9% quit during the first trimester, and 9.8% continued smoking thereafter.

**Table 1:** Characteristics of the study population by gestational diabetes mellitus (GDM) and oral glucose tolerance test (OGTT)

		GDM, *n* (%)*			Pathological OGTT, *n* (%)*			Total, *n* (%)*
		Yes	No	*p*	Yes	No	*p*	
Total		32,564 (9.90)	296,328 (90.10)		38,261 (11.63)	138,858 (42.22)		328.892
BSA, mean (SD)		1.86 (0.23)	1.72 (0.17)	< 0.01	1.85 (0.23)	1.77 (0.19)	< 0.01	1.73 (0.19)
Maternal age, mean (SD)		29.52 (5.50)	27.66 (5.19)	< 0.01	29.51 (5.46)	28.80 (4.99)	< 0.01	27.84 (5.25)
Marital status				< 0.01			< 0.01	
Single		286	1949		347	920		2235 (0.68)
Cohabiting		14.877	133.112		17.610	64.143		147,989 (45.00)
Married		17.264	159.921		20.121	73.136		177,185 (53.87)
Unknown		137	1346		183	659		1483 (0.45)
Maternal smoking				< 0.01			< 0.01	
No smoking		25.651	23.945		30.090	115.491		265,108 (80.61)
Early pregnancy		3120	22.695		3696	11.215		25,815 (7.85)
Throughout pregnancy		3225	29.005		3867	10.094		32,230 (9.80)
Unknown		568	5171		608	2058		5739 (1.74)
BMI				< 0.01			< 0.01	
Underweight, < 20		1925	48.854		2398	14.511		50,779 (15.44)
Normal weight, 20–24.9		10.443	167.643		12.666	66.804		178,086 (54.15)
Overweight, 25–29.9		9795	56.030		11.692	39.360		65,825 (20.01)
Obese, class I, 30–34.9		5940	17.301		6754	13.200		23,241 (7.07)
Obese, class II or higher, 35 +		4461	6500		4751	4983		10,961 (3.33)

*if not stated otherwise. BMI, Body-mass index

*BSA* Body surface area

### Pre-pregnancy BSA and GDM

**Fig. 1:**
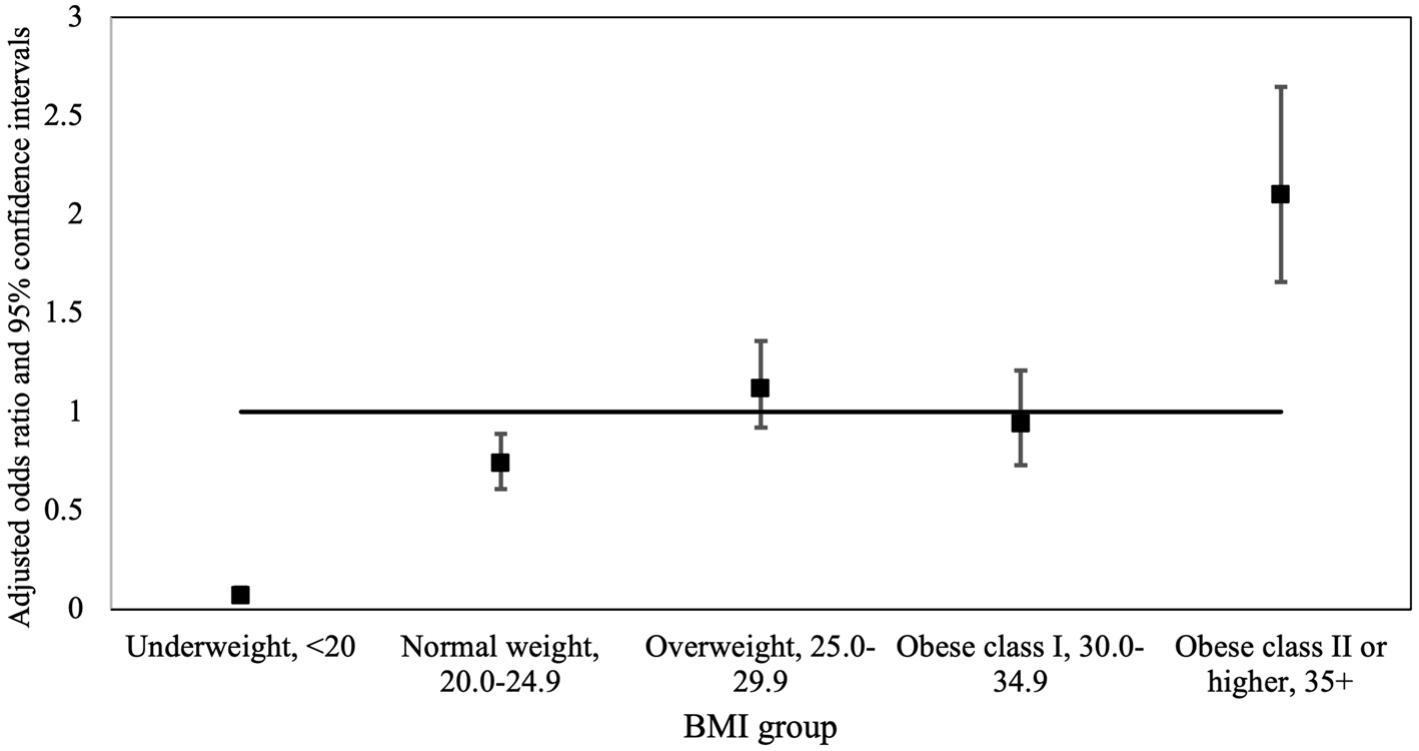
Adjusted odds ratios and 95% confidence intervals of pre-pregnancy BSA and the risk for GDM according to BMI groups

The association between the pre-pregnancy BSA and GDM was analyzed according to maternal BMI (Table 2, Fig. 1). A lower pre-pregnancy BSA predicted an increased risk for GDM among the underweight (*b* = − 2.69, SE = 0.25, OR = 0.07, 95% CI 0.04–0.11, *p* =  < 0.001) pregnant women and normal weight (*b* = − 0.30, SE = 0.10, OR = 0.74, 95% CI 0.61–0.89, *p* = 0.002), pregnant women. A similar association was not observed among the overweight (*b* = 0.11, SE = 0.10, OR = 1.12, 95% CI 0.92–1.36, *p* = 0.25) and obese class I women (*b* = − 0.06, SE = 0.13, OR = 0.94, 95% CI 0.73–1.21, *p* = 0.64). In contrast, among the obese class II or greater, a higher pre-pregnancy BSA predicted a higher risk for GDM (*b* = 0.74, SE = 0.12, OR = 2.10, 95% CI 1.66–2.65, *p* < 0.001). Smoking during the first trimester and throughout the pregnancy was a significant predictor for GDM in almost all BMI classes.

**Table 2:** Results from the logistic regression models estimating the association between the body surface area (BSA) and the risk for gestational diabetes mellitus (GDM) according to maternal body mass index (BMI)

	BMI < 20		BMI 20–24.9		BMI 25–29.9		BMI 30–34.9		BMI 35 +	
	*b*	SE	*b*	SE	*b*	SE	*b*	SE	*b*	SE
Intercept	− 1.15*	0.41	− 4.39*	0.19	− 3.80*	0.22	− 2.80*	0.31	− 3.72*	0.34
BSA	− 2.69*	0.25	− 0.30*	0.10	0.11	0.10	− 0.06	0.13	0.74*	0.12
Maternal age	0.09*	< 0.01	0.08*	< 0.01	0.06*	< 0.01	0.06*	< 0.01	0.06*	< 0.01
*Marital status*										
Single	Ref		Ref		Ref		Ref		Ref	
Cohabiting	− 0.41*	0.17	− 0.17	0.09	0.18	0.11	0.17	0.15	0.05	0.19
Married	− 0.42*	0.17	− 0.17*	0.09	0.20	0.11	0.16	0.15	0.01	0.19
*Maternal smoking*										
No smoking	Ref		Ref		Ref		Ref		Ref	
Early pregnancy	0.22*	0.09	0.34*	0.04	0.30*	0.04	0.28*	0.05	0.10	0.06
Throughout pregnancy	− 0.05	0.09	0.10*	0.04	0.16*	0.04	0.14*	0.05	0.15*	0.06

*p < 0.05. *b*, Beta;* SE* Standard error

### Pre-pregnancy BSA and OGTT

**Fig. 2:**
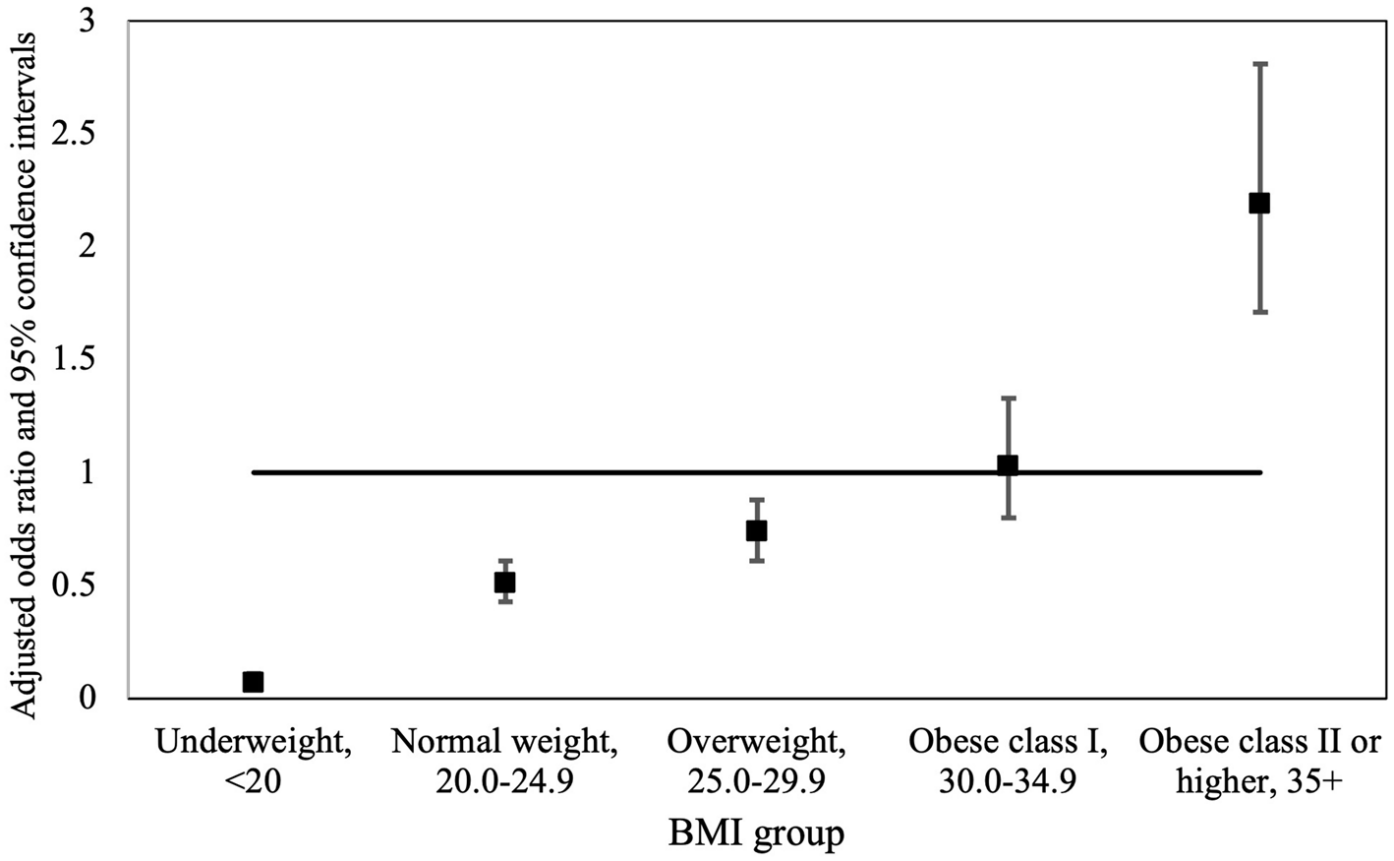
Adjusted odds ratios and 95% confidence intervals of pre-pregnancy BSA and the risk for a pathological OGTT according to BMI groups

The association between the pre-pregnancy BSA and OGTT was also analyzed according to maternal BMI (Table 3, Fig. 2). A lower pre-pregnancy BSA predicted an increased risk for pathological OGTTs among the underweight (*b* = − 2.66, SE = 0.23, OR = 0.07, 95% CI 0.04–0.11, *p* < 0.001), normal weight (*b* = − 0.67, SE = 0.09, OR = 0.51, 95% CI 0.43–0.61, *p* < 0.001), and overweight (*b* = − 0.31, SE = 0.10, OR = 0.74, 95% CI 0.61–0.88, *p* = 0.001) pregnant women. Among the obese class I women, such an association was not observed (*b* = 0.03, SE = 0.13, OR = 1.03, 95% CI 0.80–1.33, *p* = 0.79). In contrary, among the obese class II or greater, a higher pre-pregnancy BSA predicted a higher risk for pathological OGTTs (*b* = 0.79, SE = 0.13, OR = 2.19, 95% CI 1.71–2.81, *p* < 0.001). Maternal smoking was a significant predictor for pathological OGTTs in all BMI classes.

**Table 3:** Results from the logistic regression models estimating the association between the body surface area (BSA), and the risk for a pathological oral glucose tolerance test (OGTT) according to maternal body mass index (BMI)

		BMI < 20		BMI 20–24.9		BMI 25–29.9		BMI 30–34.9		BMI 35 +	
		*b*	SE	*b*	SE	*b*	SE	*b*	SE	*b*	SE
Intercept		2.41*	0.40	− 1.15*	0.19	− 2.20*	0.21	− 2.36*	0.30	− 3.25*	0.36
BSA		− 2.66*	0.23	− 0.67*	0.09	− 0.31*	0.10	0.03	0.13	0.79*	0.13
Maternal age		0.01*	< 0.01	0.03*	< 0.01	0.05*	< 0.01	0.06*	< 0.01	0.06*	< 0.01
Marital status											
Single		Ref		Ref		Ref		Ref		Ref	
Cohabiting		− 0.46*	0.17	− 0.21*	0.08	0.03	0.10	− 0.07	0.14	− 0.15	0.20
Married		− 0.46*	0.17	− 0.21*	0.08	0.07	0.10	− 0.12	0.14	− 0.22	0.20
Smoking											
No smoking		Ref		Ref		Ref		Ref		Ref	
Early pregnancy		0.24*	0.08	0.27*	0.04	0.22*	0.04	0.28*	0.05	0.12	0.07
Throughout pregnancy		0.38*	0.09	0.47*	0.04	0.37*	< 0.01	0.24*	0.05	0.30*	0.06

*p < 0.05. *b*, beta; SE, Standard error

### Sensitivity analyses

Supplementary table 1 presents the characteristics of the participants according to the combined information on GDM diagnoses and pathological OGTTs. The pattern of results in the sensitivity analyses remained significant (Supplementary Table 2).

## Discussion

To our knowledge, this is the first study to investigate the association between the maternal pre-pregnancy BSA and GDM, as well as findings of OGTTs by the BMI class. Our result showed that a lower pre-pregnancy BSA predicted an increased risk for GDM among the underweight and normal weight pregnant women, as well as an increased risk for pathological OGTTs among underweight, normal weight, and overweight pregnant women. Contrarily, a higher BSA predicted a higher risk for GDM among the women in the obese class II or greater, and a higher risk for pathological OGTTs among the women in the obese class I and obese class II or greater.

The previous studies in the general population have questioned the accurate interpretation of OGTT [8, 9]. Rehunen et al. [9] found among the 2659 participants aged 45–70 years with at least one cardiovascular risk factor but no previously diagnosed diabetes or manifested cardiovascular disease, that the height of the person was inversely associated with 2-h plasma glucose in the three lowest BMI groups, but not in the highest BMI group. Palmu et al. [8] found in the same population that the body size (assessed with BSA) had an inverse linear impact on the findings from a standardized OGTT in all categories of glucose tolerance, such as that smaller persons were more likely to be glucose intolerant than relatively larger-sized individuals. Regarding pregnant women, a meta-analysis, including ten studies by Arafa et al. [15], found that short stature was associated with a higher risk for GDM. They found that each 5-cm increase in height was associated with an approximately 20% reduction in the risk of GDM. These previous studies support the results of our study that smaller-sized women may be more susceptible to GDM.

The important question is whether these pregnant women, who may be more susceptible to a diagnosis of GDM due to their smaller body size, show more GDM-related complications than their peers who were not diagnosed with GDM or not. Chu et. al. [10] also found the inverse association between maternal height and the risk for GDM. They further analyzed the risk for GDM-related complications, including preterm births and higher birth weights. They found that the complication risk was at the same level as non-GDM women of similar height, i.e., only taller women had an increased risk of GDM-related pregnancy complications [10]. In the future, the risk for GDM-complications should be investigated in accordance with the BSA. Chu et al. [10] speculate that an artifactual GDM diagnosis due to glucose-overload among shorter women is plausible. Based on our results, we suggest that the reason behind the previously observed inverse association between the maternal height and GDM is actually the body size, i.e., body surface area, of the pregnant women, which is used to evaluate drug doses and medical indicators or estimates [11].

The question remains: What should be done to increase the accuracy of diagnosing GDM, and to prevent possible artifactual GDM diagnoses? Thus, there is a need for future studies to investigate the amount of glucose doses in the OGTT based on the size of the pregnant woman, which could provide a more specific and reliable GDM diagnosis. Furthermore, this would be important because the GDM diagnosis has a negative echo. The diagnosis is easily associated with unfavorable lifestyle factors and being overweight [16]. Pregnant women are vulnerable to weight stigma, which causes a lot of stress and negative emotions. Guilt and shame adversely affect maternal hormone levels, and impair the health of the mother and offspring in many ways. [17, 18]. Even the pregnancy-related weight stigma can potentially increase the risk of GDM and other complications during pregnancy [16, 19].

Among the general population, smoking has been found to be associated with blood glucose intolerance, impaired fasting glucose, and type two diabetes [20]. Smoking was found to be a predictor for GDM in our study. Studies on the association between smoking and GDM are conflicting [20–22]. According to a meta-analysis [22], smoking is not associated with an increased risk of GDM. However, it has been noted in the recent population-based register study [21] and prospective study [23] that smoking during pregnancy is associated with an increased risk for GDM.

### Strengths and limitations

The strength of this study includes the use of large and comprehensive national register data that included 94.5% of all primiparous women with singleton pregnancies during the study years between 2006 and 2019. Our data included information on maternal background, as well as the diagnoses of pre-pregnancy diabetes and pre-pregnancy chronic hypertension.

The main limitation of this study was that the results of OGTT were dichotomous; thus, we did not have access to specific OGTT results. Another limitation is the possibility of missing data regarding the GDM diagnoses. The prevalence of GDM/OGTT (13%) in our study population of primiparous women is lower compared to the GDM prevalence of 19% from the year 2019 in Finland [5]. The reason for this is that the prevalence of GDM has been rapidly increasing during the last few decades, and our study population includes primiparous women during the years of 2006–2019. We performed sensitivity analyses with the combined information on GDM and pathological OGTT, where the results remained the same compared to our main analyses. In addition, the Finnish health registers are shown to be reliable for research purposes [12, 13], and we used only the variables with known good quality.

## Conclusion

In conclusion, our study showed that underweight and normal weight pregnant women with a relatively smaller BSA, who otherwise had no risk factors for GDM other than, e.g., age or a close relative with type 2 diabetes, were more likely to have a pathological OGTT and receive a diagnosis of GDM. Since most pregnant women undergo the OGTT, it is particularly important that the test methods and diagnostic criteria are specific and appropriate in order to avoid false positive test results and unnecessary stigmas. Our results imply that the size of pregnant women needs to be better taken into account when diagnosing GDM.

## Supplementary Information

Below is the link to the electronic supplementary material.

Supplementary file1 (XLSX 10 kb)

Supplementary file2 (XLSX 9 kb)

## Data Availability

The data cannot be shared due to data protection regulations, but similar data can be applied from the Finnish Social and Health Data Permit Authority Findata (https://findata.fi/en/).

## Abbreviations

BSA: Body surface area
BMI: Body mass index
GDM: Gestational diabetes mellitus
OGTT: Oral glucose tolerance test
ICD: International classification of diseases
